# Development of septic polysynovitis and uveitis in foals experimentally infected with *Rhodococcus equi*

**DOI:** 10.1371/journal.pone.0192655

**Published:** 2018-02-07

**Authors:** Laura Huber, Steeve Giguère, Londa J. Berghaus, Amanda Hanafi, Sarah Vitosh-Sillman, Sarah L. Czerwinski

**Affiliations:** 1 Department of Large Animal Medicine, College of Veterinary Medicine, University of Georgia, Athens, Georgia, United States of America; 2 School of Veterinary Medicine and Biomedical Sciences, University of Nebraska, Lincoln, Nebraska, United States of America; 3 Department of Small Animal Medicine and Surgery, College of Veterinary Medicine, University of Georgia, Athens, Georgia, United States of America; East Carolina University Brody School of Medicine, UNITED STATES

## Abstract

*Rhodococcus equi* is one of the most important causes of disease in foals. Infection is typically characterized by pyogranulomatous pneumonia although extrapulmonary infections occur occasionally. Uveitis and polysynovitis have been reported in foals naturally infected with *R*. *equi* and are thought to be the result of an immune-mediated process. However, the pathogenesis of these conditions is poorly understood. The objectives of this study were to document the occurrence of uveitis and polysynovitis after experimental infection with *R*. *equi* and to determine if these disorders are the direct result of infection at these sites. Foals between 3 and 4 weeks of age were infected intratracheally with virulent *R*. *equi* using inocula of 1×10^8^ CFU (high inoculum; n = 16) or 1×10^7^ CFU (low inoculum; n = 12). Foals were monitored twice daily and necropsy was performed 14 days post-infection. Aqueous humor and synovial fluid were collected aseptically and the percentage of affected lung was calculated. The mean (± SD) percentage of affected lung was significantly higher with the high inoculum (31.8 ± 14.6%) than with the low inoculum (14.4 ± 11.4%). Fourteen of 25 foals developed uveitis and 20 of 28 foals developed polysynovitis. *R*. *equi* was cultured from the aqueous humor of 11 foals and from the synovial fluid of 14 foals. The risk of development of polysynovitis and protein concentration in the aqueous humor were significantly higher in foals that received the high inoculum. These results indicate that polysynovitis and uveitis are septic complications associated with the severity of lung disease.

## Introduction

*Rhodococcus equi* is a Gram-positive facultative intracellular pathogen that has the ability to survive and even replicate in macrophages. Infections caused by *R*. *equi* represent one of the most important causes of disease in foals. *R*. *equi* is also as a common cause of opportunistic infections in immunosuppressed people, particularly in individuals receiving chemotherapy or infected with the human immunodeficiency virus [1–3]. Pneumonia is the most common clinical manifestation of infections caused by *R*. *equi* in both species although numerous extrapulmonary disorders have been described [3, 4]. In a retrospective cohort of 150 foals with naturally acquired infections caused by *R*. *equi*, at least 1 of 39 extrapulmonary disorders were recognized in 74% of foals and detection of extrapulmonary disorders was associated with a worse prognosis [4].

Polysynovitis, characterized by effusion of multiple synovial structures and absence of lameness, occurs in approximately one fourth to one third of foals with naturally acquired *R*. *equi* infections [4, 5]. Uveitis has also been described in foals naturally infected with *R*. *equi* [4]. Detection of immunoglobulins within the synovial membrane or iris by immunofluorescence in a small number of affected foals combined with the fact that *R*. *equi* is rarely isolated from these sites at the time of diagnosis or necropsy [4–7] have led to the widespread belief that polysynovitis and uveitis are immune-mediated disorders [6–8]. However, intrabronchial challenge with a very high inoculum of virulent *R*. *equi* in one study resulted in polysynovitis in all 4 foals [9]. Culture of the synovial fluid of affected foals within a few days of the onset of synovial effusion yielded *R*. *equi* and histologic examination of the synovial membrane revealed suppurative inflammation [9]. Therefore, an alternative hypothesis is that septic polysynovitis and uveitis results from systemic infection, but that the bacteria are eventually cleared from these sites resulting in chronic non-septic inflammation at the time of diagnosis. Understanding the pathogenesis of polysynovitis and uveitis is important because immunosuppressive agents indicated for the treatment of immune-mediated disorders might be detrimental if these complications are the result of infection.

As a basis for this study, it was hypothesized that uveitis and polysynovitis are infectious processes and that their occurrences are associated with the severity of pneumonia. The objectives were to document the occurrence of uveitis and polysynovitis after experimental infection with different inocula of *R*. *equi* and to determine if *R*. *equi* can be cultured from the synovial fluid and aqueous humor.

## Materials and methods

### Preparation of *R*. *equi* for challenge

A strain of *R*. *equi* isolated from the lower respiratory tract of a foal with severe pneumonia was used in this study. The strain was confirmed to contain the virulence plasmid by amplification of *vapA* by PCR using primers described previously [10]. Bacteria were kept as frozen stabilates in individual 10 mL aliquots. The live bacterial concentration was verified to be 1 × 10^7^ colony forming units (CFU)/mL after thawing.

### Animals, intrabronchial challenge, and clinical monitoring

Twenty eight mixed breed foals were used in this study. The study was approved by the Institutional Animal Care and Use Committee of the University of Georgia (approval number A2017 02-013-Y2-A3). Adequate transfer of passive immunity was confirmed in foals at approximately 24 h of age by measurement of plasma IgG concentration using glutaraldehyde coagulation or a commercial immunoassay (Snap Foal IgG Test, Idexx Laboratories Inc., Westbrook, ME, USA). Foals were kept on pasture with their dams at a farm never used for breeding horses previously and not used to house horses in the past 10 years. Mares and foals were moved to individual stalls in an isolation facility 3 to 4 days prior to infection. Prior to infection, foals were determined to be healthy based on a thorough physical examination that included thoracic auscultation and ophthalmic examination. The mean (± SD) age at time of infection was 23 ± 2 days (range 21 to 27 days). Prior to infection, foals were sedated with 0.5 mg/kg of xylazine hydrochloride and 0.05 mg/kg of butorphanol tartrate, intravenously. The upper third of the neck was prepared aseptically. After infiltrating 2 mL of 2% lidocaine subcutaneously, a small stab incision was made through the skin and a 12 gauge, 8.9 cm long catheter was inserted in the lumen of the trachea. After removing the stylet, a 5 French polypropylene tube was cut to a length of 20 cm and inserted through the catheter for delivery of the inoculum in the trachea well rostral to the division of the 2 main bronchi. The first 16 foals received 10 mL of the inoculum (high inoculum = total dose of 1 × 10^8^ CFU) and the following 12 foals received 1 mL of the inoculum in 9 mL of sterile phosphate buffered saline (PBS) (low inoculum = total dose of 1 × 10^7^ CFU). The day of infection was designated as day 0. Baseline values for heart rate, respiratory rate, and temperature, were obtained on day 0, prior to sedation. Animals were assessed throughout the study based on twice daily complete physical examination by experienced veterinarians. At least once daily, the physical examination was performed by a veterinarian board-certified in large animal internal medicine. The physical examinations included visual inspection and palpation of the joints and heart rate, respiratory rate, and temperature recording. Criteria for euthanasia prior day 14 post-infection were: 1-decreased milk consumption for 24 hours; 2- unable or unwilling to rise with minimal vocal stimulation when entering the stall; and 3- respiratory distress for 24 hours. Respiratory distress was defined as inappropriate degree of effort to breathe based on a combined assessment of respiratory rate, rhythm, and character. Milk consumption was considered decreased if the mare’s udder was full and painful or if the foal was not observed to nurse for 24 h. One foal was euthanized one day early because of suspected decreased milk consumption. Polysynovitis was defined as presence of intra-articular effusion in more than one joint as identified by visual inspection and palpation. An ophthalmic examination was repeated on day 14 post-infection. Uveitis was defined as presence of aqueous flare, hypopyon, or hyphema.

### Post-mortem examination and sample processing

Euthanasia was performed on day 14 post-infection by intravenous administration of a lethal dose of pentobarbital and phenytoin sodium. All organs were examined macroscopically and digital images of the dorsal and ventral aspects of the lungs were obtained. The entire lung field was palpated and then cut in sections in an attempt to detect lesions not visible from the surface. Given that lesions in all affected foals were visible from the surface, images were uploaded to Photoshop (Adobe Systems Inc., San Jose, CA) for measurement of the total area of the lungs and of the area of affected lung. For each foal, results were expressed as the mean percentage of affected lungs. Lung and synovial membrane tissue from a tarsocrural joint were collected and fixed in 10% buffered formol-saline. The fixed tissues were embedded in paraffin, sectioned at 4 μm, stained with hematoxylin and eosin (H&E) and examined histologically by a pathologist unaware of inoculum size and clinical findings. Aqueous humor was collected aseptically from both eyes and synovial fluid was collected aseptically from both tarsocrural joints. Fluid samples were frozen at -80°C until processed within 2 months of collection. After thawing, aliquots (50 μL) were placed in 1 mL of brain heart infusion broth and incubated at 37°C for 24 h. Cultures were centrifuged at 16,000 × g for 5 minutes. The pellet was resuspended in 100 μL of PBS, plated on tryptic soy agar plates, and incubated 37°C for 48 h. *R*. *equi* was identified by colony morphology and PCR amplification of the *choE* gene of selected colonies from each positive culture using previously published primers [11]. Presence of the virulence plasmid was assessed by PCR amplification of *vapA*. Aqueous humor samples from a given foal were considered positive if *R*. *equi* was cultured from at least one eye. Similarly, synovial fluid samples from a given foal were considered positive if *R*. *equi* was cultured from at least one joint. Protein concentrations in aqueous humor were measured using a turbidimetric method (TPCU3, Roche Diagnostics, Indianapolis, IN, USA) and protein concentrations in synovial fluid were measured using a colorimetric assay (TP2, Roche Diagnostics) on a chemistry analyzer (Cobas c501, Roche Diagnostics) in a commercial diagnostic laboratory (Veterinary Medical Center, University of Georgia, Athens, GA, USA). Samples with protein concentrations above the analytical range were diluted as necessary. For a given foal, protein concentrations from both eyes and joints were averaged for data analysis.

### Statistical analysis

Normality of the data was assessed based on examination of histograms and normal quantile plots of residuals and using the Shapiro-Wilk test. Constant variance of the data was assessed by plotting residuals against predicted values and using Levene’s test. Heart rate, respiratory rate and temperature data were analyzed using linear mixed-effects modeling with foal modeled as a random effect and inoculum (high vs. low), day of the study, and interaction between inoculum and day modeled as fixed nominal effects. Model fit was assessed using Akaike’s information criterion values. Multiple pairwise comparisons were performed using the method of Sidak to control for family-wise type I error rates. The Student t test was used to compare continuous variables between 2 groups. Corrections for unequal variances were applied when necessary. Fisher’s exact test was used to compare dichotomous variables. Spearman correlation coefficient on rank was used to determine if there was an association between the percentage of affected lung and aqueous or synovial fluid protein concentration. Cox proportional hazard regression was used to compare the rate of development of polysynovitis between inocula. The proportional hazard assumption was tested graphically using log-log plots and statistically on the basis of Schoenfeld residuals. Kaplan–Meier curves were generated to display development of polysynovitis over time for the 2 inocula (high vs low). For all analyses, significance was set at *P* < 0.05.

## Results

**Fig 1:**
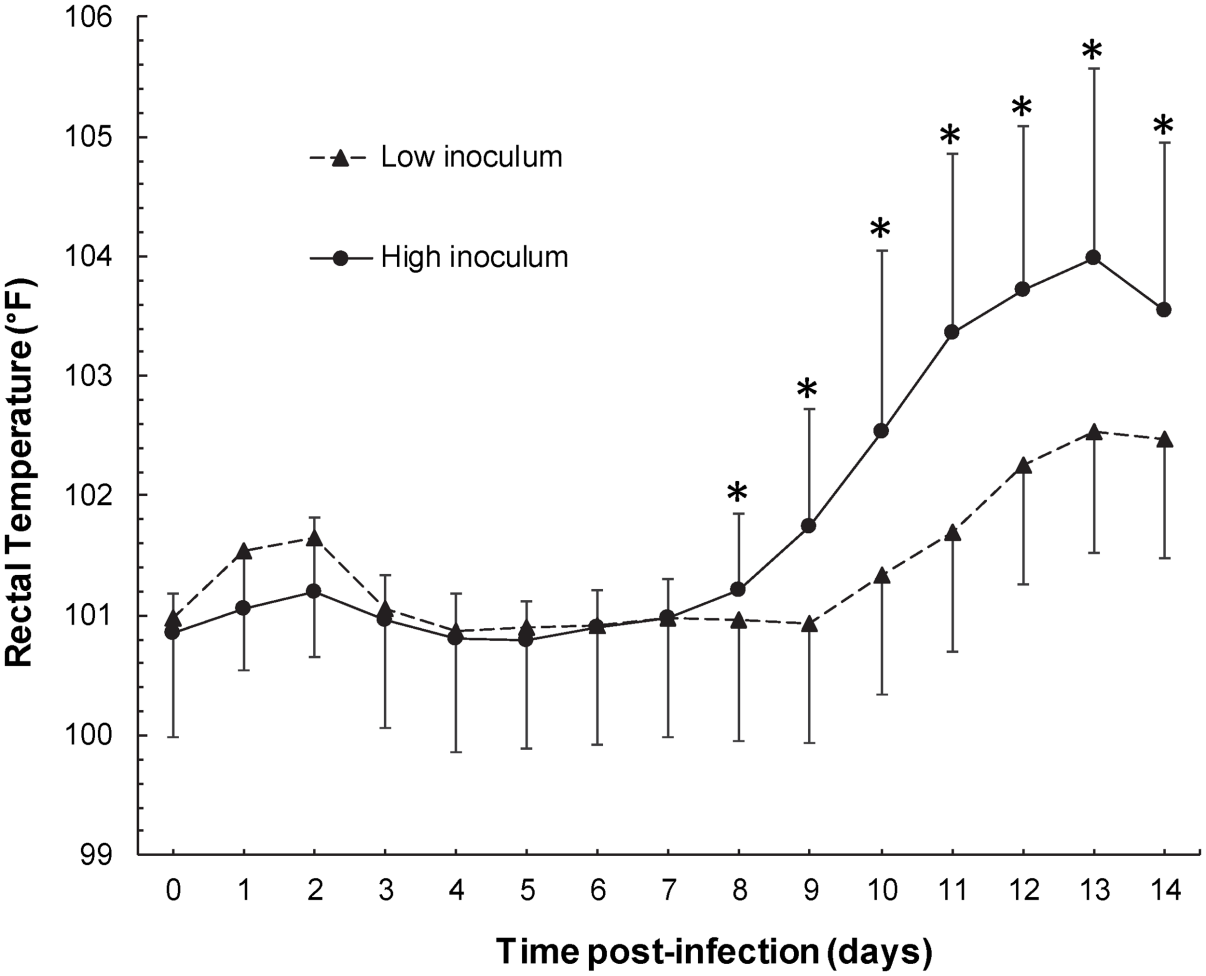
Mean (±SD) rectal temperature of foals experimentally infected with virulent *R*. *equi*. Foals were infected with a high inoculum (1 × 10^8^ CFU; n = 16) or a low inoculum (1 × 10^7^ CFU; n = 12). *Indicates a significantly higher rectal temperature relative to day 0 and relative to the temperature of foals infected with the low inoculum.

**Fig 2:**
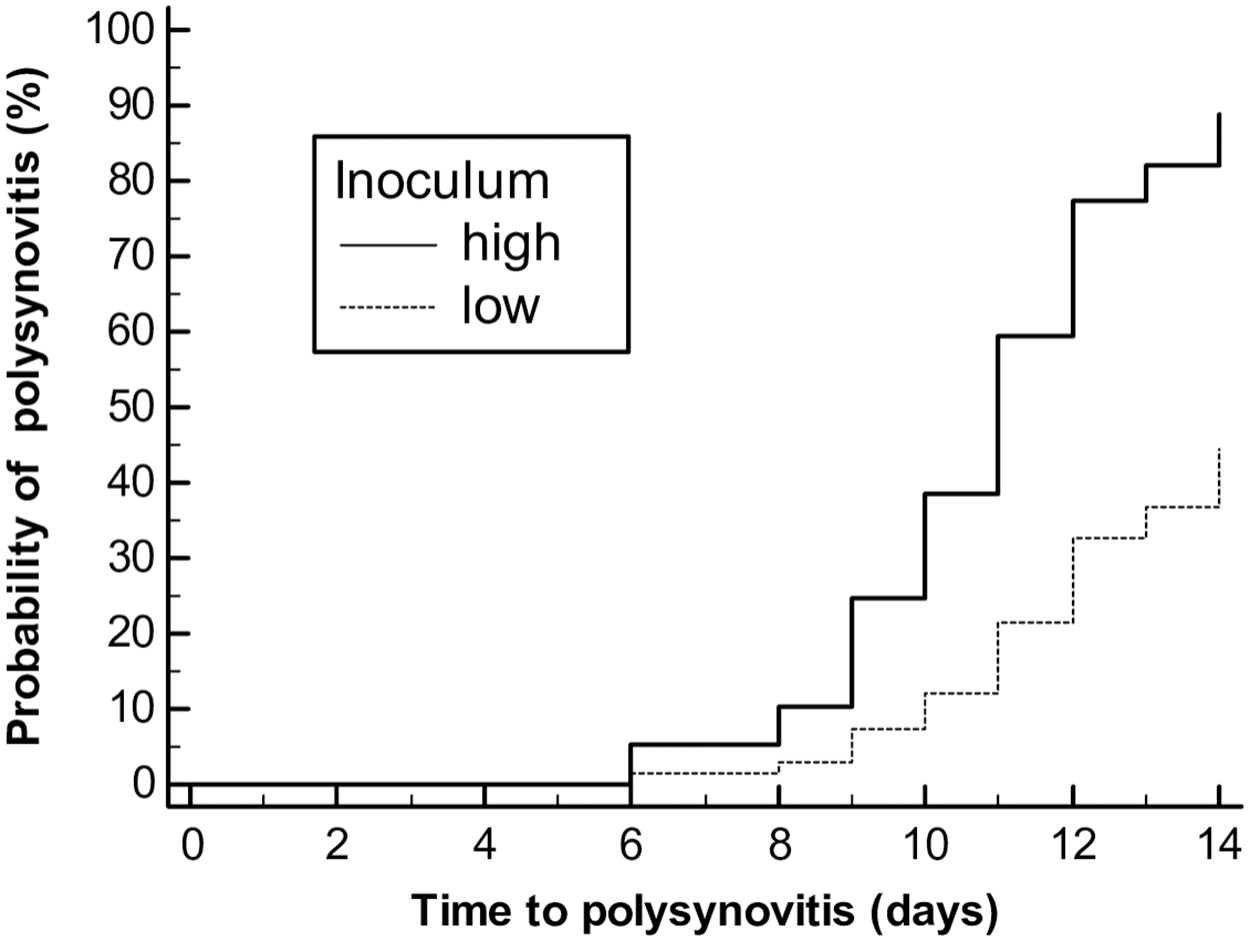
Kaplan-Meier curve of the cumulative probability of developing polysynovitis in foals infected intratracheally with a high inoculum (1 × 10^8^ CFU; n = 16) or a low inoculum (1 × 10^7^ CFU; n = 12) of virulent *R*. *equi*.

There was a significant effect of inoculum (P < 0.001), day of post-infection (P < 0.001) and a significant interaction between inoculum and day of infection (P = 0.001) on rectal temperature. Beginning 9 days post-infection, foals infected with the high inoculum developed significantly higher rectal temperatures compared to their baseline values and to that of foals infected with the low inoculum (Fig 1). Heart rate and respiratory rate did not increase significantly relative to baseline and were not significantly different between the high and low inocula. Twenty of 28 foals (71%) developed polysynovitis of the fetlocks, stifles, carpi and tarsocrural joints. There was no apparent lameness in affected foals. The risk of development of polysynovitis was reduced by approximately 66.4% (hazard ratio = 0.336; 95% CI = 0.138 to 0.817; P = 0.016) in foals infected with the low inoculum relative to foals infected with the high inoculum (Fig 2). Fourteen of 25 foals (56%) subjected to an ophthalmic examination on day 14 post-infection had uveitis as defined by the presence of aqueous flare. Two of the 14 foals also had bilateral hypopyon. There was a strong and statistically significant (P < 0.001) association between polysynovitis and uveitis with all 14 foals with uveitis also having polysynovitis. Only 3 foals had polysynovitis without clinically detectable uveitis. Other clinical signs detected occasionally included intermittent cough (n = 4) and mild self-limiting diarrhea (n = 9).

**Fig 3:**
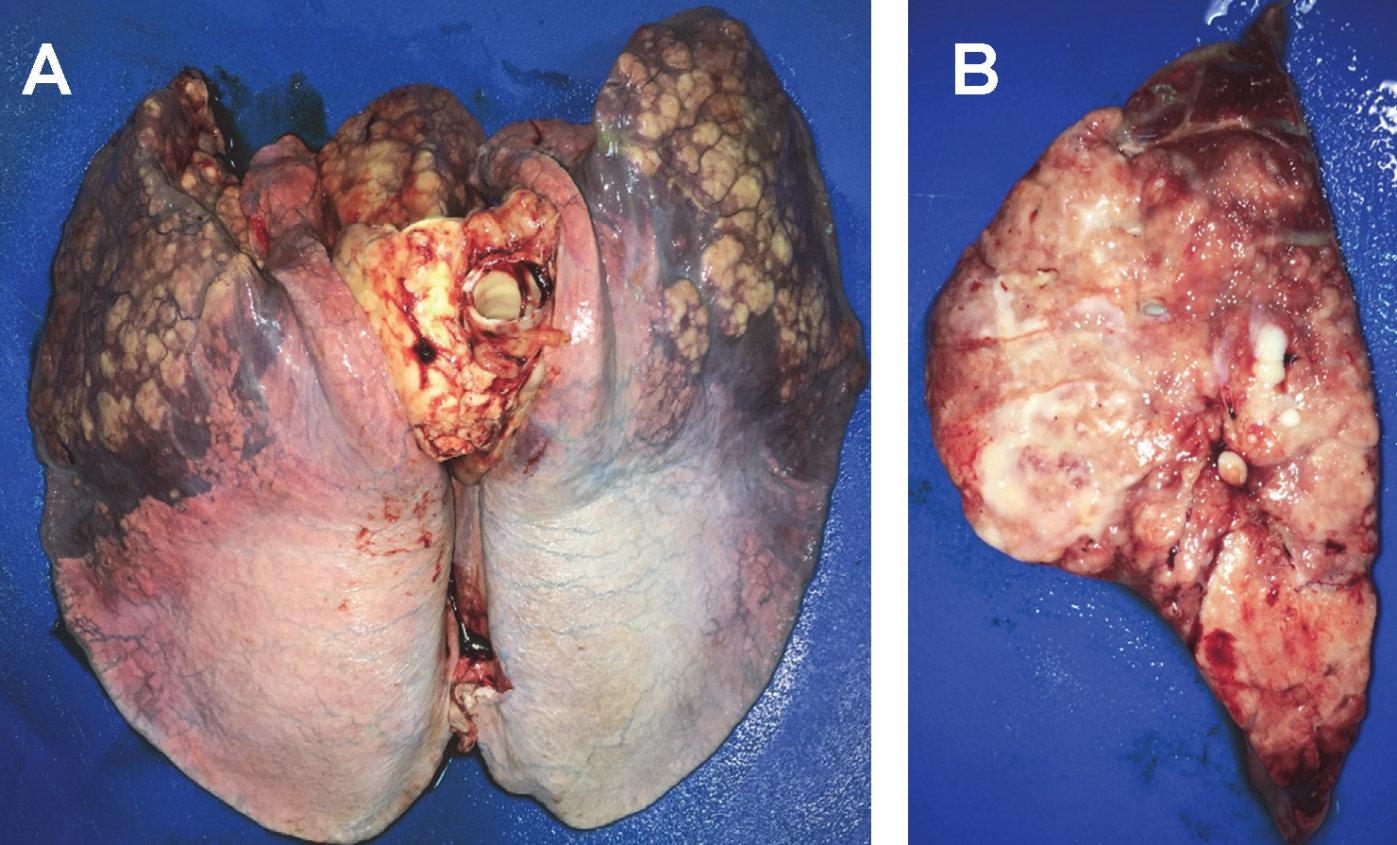
Macroscopic pathological findings in a foal infected intratracheally with of virulent *R*. *equi* (1 × 10^8^ CFU) and euthanized 14 days post-infection. (A) Severe bilateral consolidation of the cranio-ventral lungs. (B) Cross section of a cranio-ventral lung lobe showing multiple coalescing nodular areas of pulmonary consolidation and purulent exudate.

**Fig 4:**
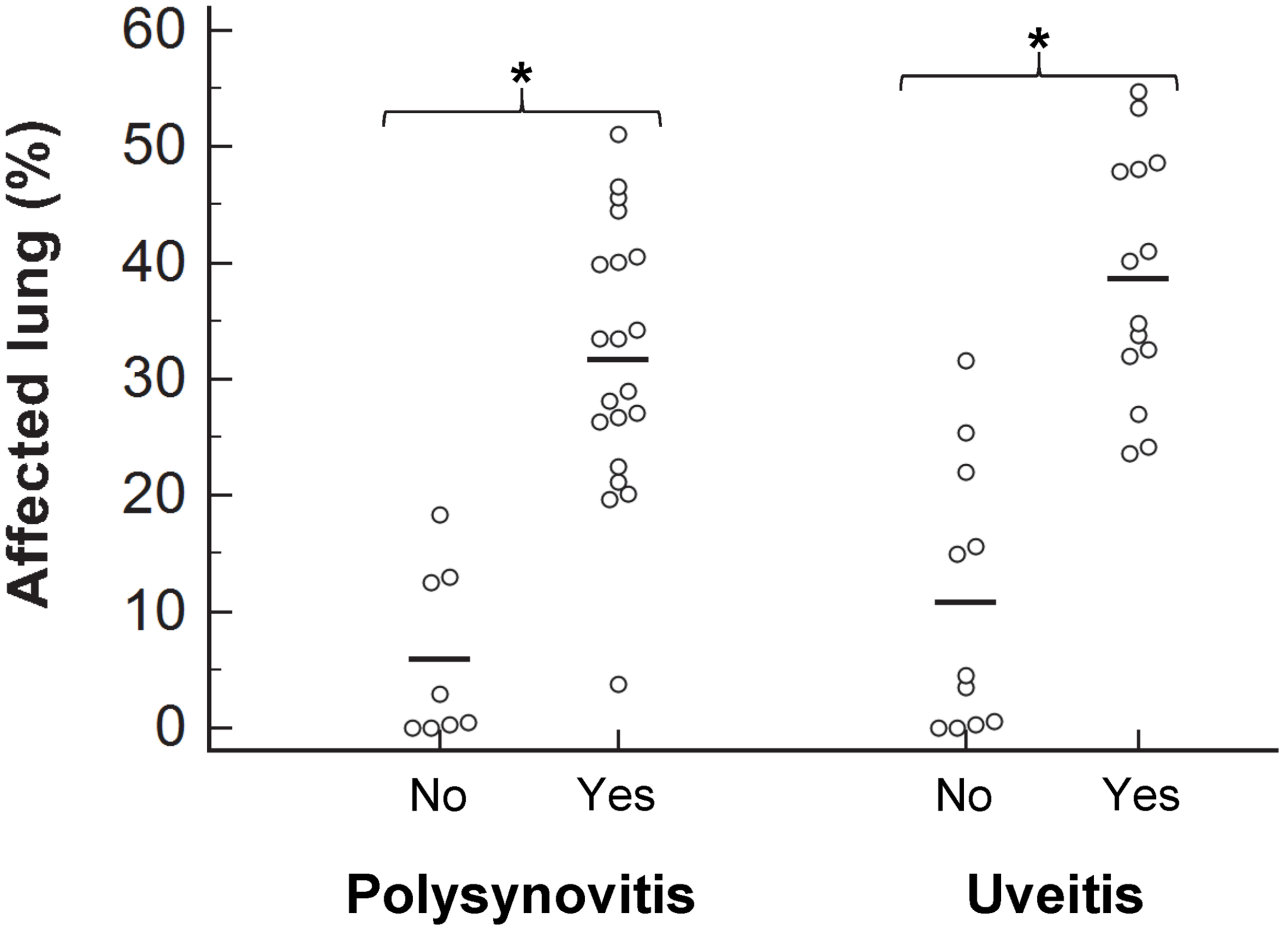
Comparison of the mean percentage of affected lung in 28 foals with or without clinical evidence of uveitis or polysynovitis (yes or no) after intratracheal infection with virulent *R*. *equi*. Each symbol represents an individual foal and horizontal lines represent the mean. *Indicates a statistically significant difference between groups (P < 0.001).

Twenty-six of 28 foals had pneumonia ranging from small discrete granulomas or abscesses to severe consolidation of the cranio-ventral and accessory lobes (Fig 3A). On cut section, the consolidated parenchyma comprised multiple coalescing, tan-colored nodular lesions some of which containing central areas of caseous necrosis (Fig 3B). Two foals had normal lungs macroscopically. The bronchial lymph nodes of all foals with pulmonary lesions were enlarged. Histopathology of affected lungs revealed severe, diffuse, chronic, pyogranulomatous pneumonia with macrophages and giant cells containing numerous bacteria. The mean (± SD) percentage of affected lung in foals infected with the high inoculum (31.8 ± 14.6%; range 0 to 51.1%) was significantly (P = 0.002) higher than that of foals infected with the low inoculum (14.4 ± 11.4%; range 0 to 33.5%). The percentage of affected lung was significantly higher for foals with clinically detectable polysynovitis and uveitis relative to that of unaffected foals (Fig 4).

**Fig 5:**
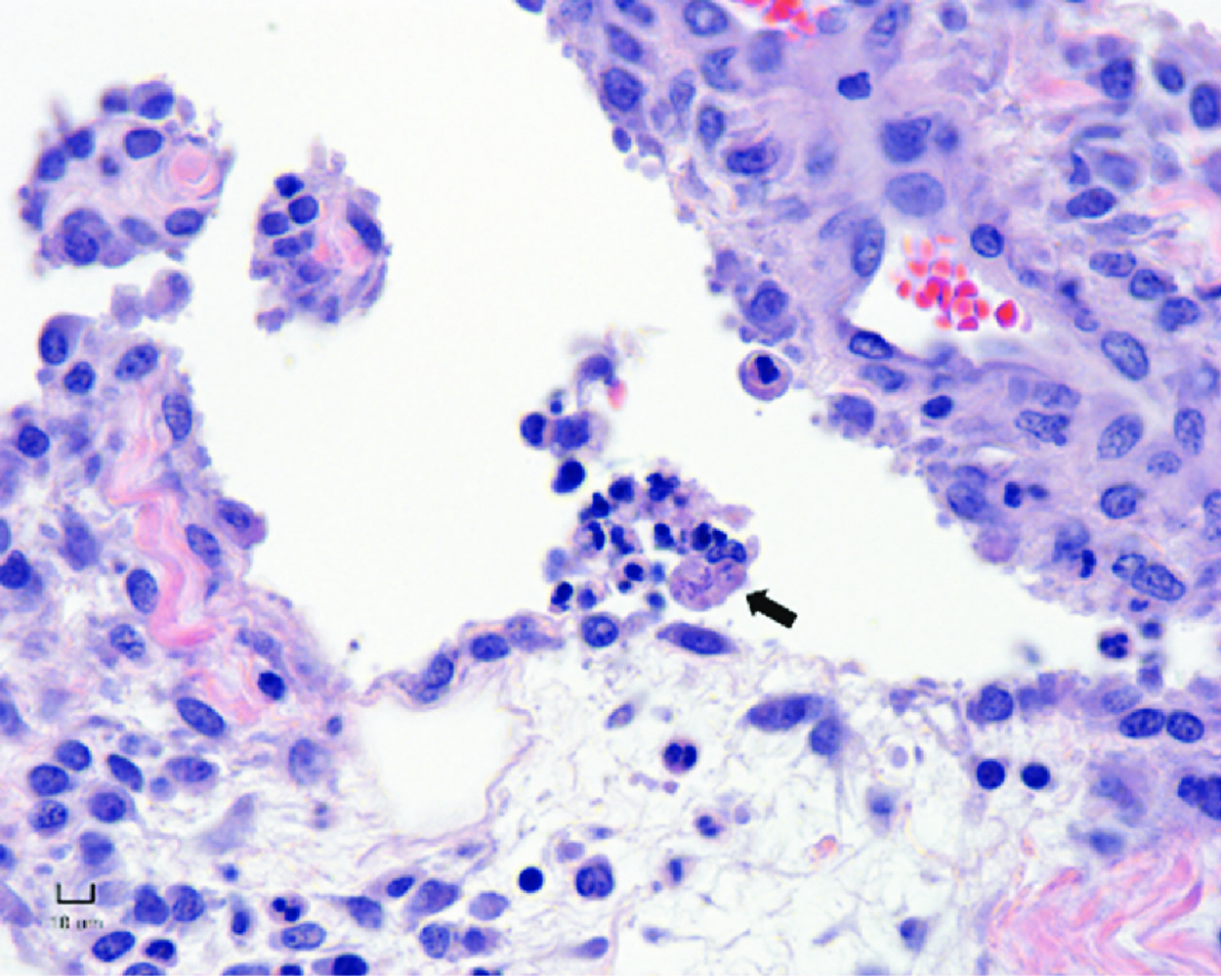
Synovitis with hyperplasia of synoviocytes, suppurative inflammation, and fibrinocellular joint exudate including macrophage (arrow) containing multiple intracytoplasmic, 2 x 3 μm coccobacilli in a foal that developed polysynovitis after intratracheal infection with virulent *R*. *equi*. Hematoxylin and eosin, bar = 10 μm.

Culture of aqueous humor yielded *R*. *equi* positive for *vapA* in 11 of 25 foals (5 with uveitis and 6 without). Culture of the synovial fluid yielded *R*. *equi* positive for *vapA* in 14 of 28 foals (12 with polysynovitis and 2 without). Aqueous humor and synovial fluid samples from the 2 foals with normal lungs did not yield *R*. *equi*. Histopathology of the synovial membrane was available for 22 foals. Of those, 6 were normal or had only mild hyperplasia of synoviocytes and all 6 samples were from foals without polysynovitis. Samples from 16 foals revealed synovitis characterized by marked hyperplasia of synoviocytes, variable proliferation of stromal cells in the synovium, moderate edema, and diffuse lymphohistiocytic and suppurative infiltrates. Fibrinosuppurative material was occasionally adhered to the synovial membrane. Macrophages containing many intracytoplasmic, 2 x 3 μm coccobacilli were identified in synovial membrane samples from 10 of the 16 foals (Fig 5). All 10 foals had polysynovitis but only 5 of these 10 foals had positive culture for *R*. *equi* in the synovial fluid.

Aqueous humor total protein concentration was significantly higher in foals with clinically detectable uveitis and infected with the high inoculum than in foals without clinically detectable uveitis or infected with the low inoculum, respectively (Table 1). There was a strong positive correlation (*r*_*s*_ = 0.80; P < 0.001) between the percentage of affected lung and aqueous humor total protein concentration. Aqueous humor total protein concentration was significantly higher in foals with uveitis than in foals without uveitis (Table 1). There was a moderate but statistically significant positive correlation (*r*_*s*_ = 0.55; P = 0.003) between the percentage of affected lung and synovial fluid total protein concentration.

**Table 1:** Comparison of mean (± SD) total protein concentration in aqueous humor and synovial fluid between inocula (high vs low) and presence or absence of clinically detectable polysynovitis or uveitis in 28 foals experimentally infected with *R*. *equi*.

Sample	Uveitis/polysynovitis		Inoculum	
	Present	Absent	High	Low
Aqueous humor (mg/dL)	1,578 ± 707^a^	103 ± 95	1,423 ± 971^c^	393 ± 432
Synovial fluid (g/dL)	3.0 ± 0.5^b^	1.7 ± 0.4	2.8 ± 0.8	2.5 ± 0.7

^a^ Significantly higher than in foals without uveitis (P < 0.001).

^b^ Significantly higher than in foals without polysynovitis (P < 0.001).

^c^ Significantly higher than in foals infected with the low inoculum (P = 0.003).

## Discussion

The present study documents the occurrence of polysynovitis and uveitis in a high proportion of foals experimentally infected with virulent *R*. *equi*. The culture of virulent *R*. *equi* from aqueous humor and synovial fluid indicates that these extrapulmonary disorders are septic and not immune-mediated processes. Multiple studies have described development of pneumonia in foals after experimental infection with 10^3^ to 10^10^ CFU of virulent *R*. *equi* by instillation into the airways or by nebulization. However, uveitis had not been described in foals experimentally infected with *R*. *equi* and polysynovitis was only described in 4 foals after infection with 10^9^ CFU [9]. The reasons for the lack of detection or reporting of polysynovitis and uveitis in prior experimental infections are unknown but these complications would be easily missed without thorough ocular examination and palpation of the joints. In the present study, foals with polysynovitis did not have apparent lameness and foals with uveitis did not have obvious signs of ocular disease such as blepharospasm or ocular discharge. The clinical diagnosis of polysynovitis and uveitis is somewhat subjective and might vary depending on the experience or expertise of the clinician. Therefore we relied also on synovial fluid and aqueous humor protein concentration and histopathology by a blinded pathologist to more objectively detect and quantify inflammation at these sites.

In this study the association between clinically detectable polysynovitis and positive culture for *R*. *equi* was not perfect since eight foals with polysynovitis had negative cultures. Histopathology of the synovial membrane was performed in 6 of these 8 foals and suppurative inflammation with intracellular coccobacilli was observed in 5 foals. Failure to culture bacteria from synovial fluid in cases of septic arthritis is common in horses. In a retrospective study of horses with naturally occurring septic arthritis culture was negative in approximately 24% of cases [12]. Similarly, 9 of 14 foals with uveitis had negative culture. The 2 foals with the most chronic uveitis as evidenced by hypopyon and fibrin in the anterior chamber had negative cultures. Similarly, *R*. *equi* is rarely isolated from the aqueous humor of naturally infected foals which typically have chronic disease at the time of sampling [4]. These findings suggest that culture might be less likely to be positive in more chronic cases of uveitis. Although *R*. *equi* is fairly resilient to temperature changes, the possibility that the sensitivity of culture was decreased by prior freezing of the samples cannot be excluded. Sensitivity of detection of *R*. *equi* in aqueous humor and synovial fluid might have been improved by PCR amplification. In adult horses with recurrent uveitis, PCR for amplification of *Leptospira* DNA in aqueous humor is much more sensitive than culture [13]. Similarly PCR is more sensitive than culture for the detection of bacteria in synovial fluid samples from horses with clinical evidence of septic arthritis [14]. In the present study we elected to rely only on culture to detect the presence of live bacteria.

Conversely, 2 foals without clinical evidence of polysynovitis and 6 foals without clinically apparent uveitis had positive cultures for *R*. *equi*. Some of these foals likely had subclinical uveitis as evidenced by increased protein concentrations in aqueous humor. Aqueous humor protein concentration has been positively correlated to aqueous flare measurements using flaremetry [15, 16], which would have been a more sensitive and objective indicator of uveitis when compared to clinical examination. It is also possible that some foals were euthanized prior to development of uveitis or polysynovitis. Finally, it is also possible that a small proportion of foals with positive cultures for *R*. *equi* in synovial fluid or aqueous humor might clear the bacteria without necessarily developing uveitis or polysynovitis.

The detection of *R*. *equi* in joint fluid and aqueous humor from multiple foals indicates hematogenous dissemination of *R*. *equi*. Intermittent or persistent bacteremia with *R*. *equi* might be more common than previously recognized given that blood cultures are not routinely performed in foals with pneumonia caused by *R*. *equi*. In one study, 6 of 10 foals with naturally acquired pneumonia had positive blood cultures [17]. More recently, *R*. *equi* was isolated from the blood of 11 of 19 foals and foals with positive blood culture results were less likely to survive than foals that were culture-negative [4]. Blood culture is also the most sensitive means of diagnosis in people infected with this *R*. *equi* [2]. Unfortunately, blood cultures were not performed in this study. Therefore, it is not possible to know when bacteremia occurred relative to infection and to development of polysynovitis or uveitis.

One of the limitations of this study is the lack of an uninfected control group. Foals raised at farms endemic for infections caused by *R*. *equi* commonly have subclinical pulmonary disease. The inclusion of a large number of uninfected control foals would have been helpful to document absence of naturally acquired pulmonary lesions and *R*. *equi* in lung tissue. The nature of the lesions observed (severe ventral consolidation with a clear demarcation between healthy and affected tissue), the high frequency of uveitis and polysynovitis, and the consistent incubation period are all consistent with a single exposure to a relatively heavy inoculum of *R*. *equi* and would be unheard of for natural infection especially at a farm never used to breed horses and raise foals in the past. Ultimately, the novel and important finding of this study is that polysynovitis and uveitis associated with pneumonia caused by *R*. *equi* are septic in nature and not just the result of an immune mediated process. Even in the unlikely hypothetical scenario where one or more foals were naturally infected with *R*. *equi* while at pasture, the conclusions that development of uveitis and polysynovitis is associated with the severity of pulmonary disease and septic in nature would be unchanged. Therefore, it was decided that the sacrifice of multiple healthy foals was not justified for ethical reasons.

The strong association between the presence and severity of uveitis and polysynovitis and the severity of lung lesions emphasize the need to look for a primary infectious process in foals diagnosed with these conditions. Knowledge of the pathogenesis of polysynovitis and uveitis has important implications regarding therapy. The use of systemic corticosteroids has been recommended for foals with polysynovitis and uveitis due to the belief that these extrapulmonary disorders are non-septic and immune-mediated [8, 18]. The septic nature of uveitis and polysynovitis and their strong association with the severity of pulmonary disease indicates that additional studies are required before systemic corticosteroids are recommended for the treatment of these conditions. The ability to reproduce these extrapulmonary disorders experimentally in a high proportion of foals would make assessment of various therapies possible in future studies.

## Supporting information

S1 Table Mean daily rectal temperature, heart rate and respiratory rate of foals experimentally infected with virulent *R*. *equi*. Foals were infected with a high inoculum (1 × 10^8^ CFU; n = 16) or a low inoculum (1 × 10^7^ CFU; n = 12).
(PDF)

S2 Table Occurrence of uveitis or polysynovitis, percentage of affected lung, culture results, and fluid protein concentrations. Foals were infected with a high inoculum (1 × 10^8^ CFU; n = 16) or a low inoculum (1 × 10^7^ CFU; n = 12) of virulent *R*. *equi*.
(PDF)
