# Supplementary material for: Development of septic polysynovitis and uveitis in foals experimentally infected with Rhodococcus equi
Source: PLoS One. 2018 Feb 7;13(2):e0192655. doi: 10.1371/journal.pone.0192655 (PMC5802921; doi:10.1371/journal.pone.0192655)
Supplement: S1 Table — Foals were infected with a high inoculum (1 × 10^8^ CFU; n = 16) or a low inoculum (1 × 10^7^ CFU; n = 12). (PDF) [file pone.0192655.s001.pdf]

S1 Table. Mean daily rectal temperature, heart rate and respiratory rate of foals experimentally infected with virulent *R. equi*. Foals were infected with a high inoculum (1 × 10^8 CFU; n=16) or a low inoculum (1 × 10^7 CFU; n=12).

Foal id	Challenge dose	Study day	Mean rectal temperature (F)	Mean heart rate (/min)	Mean respiratory rate (/min)
77	high	0	101.1	104	90
77	high	1	101.15	104	30
77	high	2	101.4	90	34
77	high	3	100.75	90	24
77	high	4	101.2	96	46
77	high	5	101.45	110	38
77	high	6	100.95	93	35
77	high	7	101.25	100	52
77	high	8	101.65	97	38
77	high	9	101.25	90	37
77	high	10	103.15	78	43
77	high	11	103.25	90	40
77	high	12	104.6	98	36
77	high	13	104.75	90	26
77	high	14	104.2	104	48
78	low	0	100.8	100	34
78	low	1	101.05	108	38
78	low	2	101.25	94	42
78	low	3	100.9	92	32
78	low	4	100.65	88	34
78	low	5	101.2	98	50
78	low	6	100.85	98	44
78	low	7	101.25	88	42
78	low	8	101.3	93	40
78	low	9	100.6	88	44
78	low	10	102.35	90	40
78	low	11	103.8	80	44
78	low	12	104.95	86	36
78	low	13	104.6	114	44
78	low	14	103.2	140	32
79	high	0	100.6	108	42
79	high	1	101.45	108	42
79	high	2	101.15	104	40
79	high	3	100.95	96	38
79	high	4	101.45	98	46

79	high	5	101.1	96	42
79	high	6	100.7	94	38
79	high	7	101.85	84	38
79	high	8	102	86	38
79	high	9	103.7	92	38
79	high	10	104.55	110	52
79	high	11	105.1	102	56
79	high	12	104.7	108	54
79	high	13	105.45	87	56
79	high	14	102.4	128	56
80	high	0	100.5	80	46
80	high	1	100.7	83	28
80	high	2	100.5	74	44
80	high	3	100.6	74	38
80	high	4	100.65	86	42
80	high	5	101.05	97	36
80	high	6	101.2	83	56
80	high	7	100.7	100	36
80	high	8	102	93	48
80	high	9	101.95	80	56
80	high	10	103.1	83	48
80	high	11	103.25	98	44
80	high	12	103.9	106	50
80	high	13	104.15	110	48
80	high	14	104.2	100	48
82	high	0	101.1	100	58
82	high	1	101.5	89	36
82	high	2	101.25	94	50
82	high	3	100.95	100	42
82	high	4	100.9	92	42
82	high	5	100.7	90	44
82	high	6	100.85	76	40
82	high	7	100.85	80	38
82	high	8	100.85	82	36
82	high	9	102.35	74	50
82	high	10	103.7	93	48
82	high	11	104.65	88	44
82	high	12	104.75	104	48
82	high	13	104.15	98	56
82	high	14	103.9	100	48
84	high	0	100.45	80	50
84	high	1	100	86	50
84	high	2	100.7	56	56

84	high	3	100.2	54	58
84	high	4	100.7	58	50
84	high	5	100.7	60	54
84	high	6	100.2	68	44
84	high	7	100.7	60	40
84	high	8	101.2	80	38
84	high	9	101.1	90	53
84	high	10	102	60	44
84	high	11	105.1	50	42
84	high	12	104.5	54	78
84	high	13	105.6	60	76
84	high	14	103.8	112	48
87	high	0	100.9	98	79
87	high	1	101.15	98	60
87	high	2	101.85	93	78
87	high	3	101.2	94	50
87	high	4	100.7	91	46
87	high	5	100.7	96	62
87	high	6	101.1	88	52
87	high	7	100.95	90	42
87	high	8	101.3	92	38
87	high	9	102.1	83	40
87	high	10	103.55	94	40
87	high	11	104.85	92	56
87	high	12	104.95	102	40
87	high	13	104.4	96	42
87	high	14	103.9	100	64
88	low	0	101.2	106	56
88	low	1	101.25	112	38
88	low	2	101.5	122	42
88	low	3	101.45	116	42
88	low	4	100.95	102	36
88	low	5	100.95	94	42
88	low	6	101.2	90	38
88	low	7	101.35	94	44
88	low	8	101.15	96	30
88	low	9	101.4	76	38
88	low	10	102	84	42
88	low	11	103.55	118	60
88	low	12	104.3	118	62.5
88	low	13	105.45	127	82
88	low	14	106.2	100	76
89	high	0	101.3	98	52

89	high	1	101.05	104	64
89	high	2	102.75	124	66
89	high	3	101.6	112	60
89	high	4	101.05	106	43
89	high	5	100.65	120	48.5
89	high	6	100.9	110	48
89	high	7	100.6	110	46
89	high	8	101.1	114	46
89	high	9	101.05	116	40
89	high	10	101.4	110	46
89	high	11	103.2	90	54
89	high	12	104.8	112	48
89	high	13	103.25	88	22
89	high	14	103.9	120	32
90	high	0	100.6	90	32
90	high	1	101.3	52	20
90	high	2	100.9	78	38
90	high	3	100.6	78	40
90	high	4	100.15	76	28
90	high	5	100.35	80	23
90	high	6	101.2	78	28
90	high	7	100.95	78	36
90	high	8	100.15	78	28
90	high	9	100	78	34
90	high	10	100.1	78	38
90	high	11	100.6	76	40
90	high	12	100.2	76	38
90	high	13	100.3	66	34
90	high	14	100	112	40
91	low	0	100.5	86	38
91	low	1	100.75	94	42
91	low	2	102.05	86	38
91	low	3	100.8	88	44
91	low	4	100.8	86	52
91	low	5	100.35	90	44
91	low	6	100.85	81	36
91	low	7	100.35	78	42
91	low	8	100.85	82	38
91	low	9	100.95	77	54
91	low	10	100.7	78	44
91	low	11	100.15	82	40
91	low	12	101.15	78	38
91	low	13	100.4	76	42

91	low	14	101	72	36
92	low	0	101.25	90	48
92	low	1	101.55	92	58
92	low	2	101.85	94	48
92	low	3	101.35	86	42
92	low	4	100.7	106	50
92	low	5	101.3	92	53
92	low	6	100.75	84	52
92	low	7	101	90	54
92	low	8	100.8	106	48
92	low	9	100.7	94	40
92	low	10	100.45	92	38
92	low	11	100.9	80	44
92	low	12	101	90	42
92	low	13	101.65	92	50
92	low	14	102.4	92	40
93	high	0	101.05	88	38
93	high	1	101.05	88	42
93	high	2	101.5	86	42
93	high	3	101.05	84	42
93	high	4	100.6	81	42
93	high	5	100.8	90	42
93	high	6	100.9	75	50
93	high	7	100.75	86	50
93	high	8	101.3	86	46
93	high	9	102.15	88	46
93	high	10	103.8	84	64
93	high	11	104	90	58
93	high	12	103.65	94	52
93	high	13	104.75	94	48
93	high	14	105.3	112	36
94	high	0	100.45	106	48
94	high	1	100.7	116	58
94	high	2	100.7	125	52
94	high	3	101.3	88	62
94	high	4	100.65	98	43
94	high	5	100.55	98	44
94	high	6	100.5	85	42
94	high	7	100.4	90	42
94	high	8	101.2	88	46
94	high	9	102.4	78	54
94	high	10	103.35	92	50
94	high	11	104.05	84	58

94	high	12	103.7	80	48
94	high	13	103.05	94	44
94	high	14	104.4	80	40
96	low	0	100.65	82	46
96	low	1	100.85	83	34
96	low	2	100.25	82	34
96	low	3	100.75	84	36
96	low	4	100.65	80	44
96	low	5	100.55	80	38
96	low	6	100.95	74	34
96	low	7	100.95	76	30
96	low	8	100.95	72	44
96	low	9	100.6	84	38
96	low	10	100.9	86	36
96	low	11	102.2	80	36
96	low	12	103.8	104	50
96	low	13	105.05	96	62
96	low	14	103.8	100	64
97	low	0	101.05	98	28
97	low	1	101.3	88	38
97	low	2	101.1	76	42
97	low	3	100.5	88	38
97	low	4	101.25	88	40
97	low	5	101.15	72	32
97	low	6	100.7	84	40
97	low	7	101.2	84	40
97	low	8	100.85	80	38
97	low	9	100.9	70	38
97	low	10	101.65	74	40
97	low	11	102.8	98	44
97	low	12	103.75	122	50
97	low	13	104.55	100	52
97	low	14	104.5	52	52
98	high	0	101.25	84	48
98	high	1	101.85	102	40
98	high	2	101.3	82	34
98	high	3	100.45	84	40
98	high	4	101.4	96	38
98	high	5	100.45	98	36
98	high	6	100.95	88	46
98	high	7	101.15	86	38
98	high	8	100.65	78	38
98	high	9	101.25	94	46

98	high	10	100.15	86	38
98	high	11	101.25	84	36
98	high	12	102.55	88	40
98	high	13	102.15	96	44
98	high	14	102.6	100	48
99	low	0	100.65	78	44
99	low	1	102.4	80	38
99	low	2	102.7	102	38
99	low	3	101.1	78	36
99	low	4	101.1	82	38
99	low	5	100.55	74	44
99	low	6	100.55	76	36
99	low	7	100.75	64	32
99	low	8	100.7	76	40
99	low	9	100.65	72	46
99	low	10	100.95	64	38
99	low	11	100.85	68	46
99	low	12	101.1	72	40
99	low	13	101.3	72	46
99	low	14	100.5	60	52
100	low	0	101.15	100	44
100	low	1	101.1	92	48
100	low	2	101.45	90	46
100	low	3	100.55	88	184
100	low	4	100.65	92	38
100	low	5	100.7	80	32
100	low	6	100.85	88	38
100	low	7	101	84	36
100	low	8	101	84	36
100	low	9	101.5	90	40
100	low	10	101.65	88	36
100	low	11	101.2	84	38
100	low	12	101.75	80	36
100	low	13	102.7	90	46
100	low	14	103.3	100	40
101	low	0	101.2	90	56
101	low	1	102.95	94	40
101	low	2	101.2	82	36
101	low	3	101.15	82	40
101	low	4	100.8	80	30
101	low	5	100.85	84	38
101	low	6	101	78	38
101	low	7	100.85	62	32

101	low	8	100.8	82	30
101	low	9	101	78	44
101	low	10	100.75	72	42
101	low	11	100.95	70	38
101	low	12	100.75	76	42
101	low	13	100.75	76	34
101	low	14	100.8	76	36
102	low	0	101.05	84	22
102	low	1	101.85	82	18
102	low	2	102.2	78	30
102	low	3	101.3	70	26
102	low	4	100.4	68	22
102	low	5	100.35	78	20
102	low	6	100.7	76	28
102	low	7	100.45	82	30
102	low	8	100.9	72	24
102	low	9	99.95	72	26
102	low	10	100.75	60	26
102	low	11	100	66	26
102	low	12	100.9	68	22
102	low	13	101	66	34
102	low	14	101.5	64	32
103	high	0	101.05	102	38
103	high	1	101.35	100	42
103	high	2	101.8	96	38
103	high	3	101.5	90	44
103	high	4	100.7	93	48
103	high	5	101	90	40
103	high	6	101.15	84	34
103	high	7	101	98	32
103	high	8	100.7	80	38
103	high	9	100.8	82	30
103	high	10	100.55	82	40
103	high	11	100.95	86	38
103	high	12	101.1	86	50
103	high	13	100.85	86	38
103	high	14	101.3	76	36
104	high	0	101.15	110	58
104	high	1	101.4	116	66
104	high	2	100.4	114	46
104	high	3	101.15	106	51
104	high	4	100.2	104	56
104	high	5	100.4	98	48

104	high	6	100.7	98	48
104	high	7	100.9	90	49
104	high	8	100.85	98	44
104	high	9	101.9	94	48
104	high	10	103.85	90	54
104	high	11	103.45	88	40
104	high	12	103.15	88	36
104	high	13	104.8	84	38
104	high	14	103.4	100	40
105	low	0	100.95	86	70
105	low	1	101.15	82	34
105	low	2	101.15	88	56
105	low	3	101.25	82	48
105	low	4	101.1	84	48
105	low	5	101.05	80	38
105	low	6	101.15	78	40
105	low	7	101.05	82	48
105	low	8	101.2	82	48
105	low	9	101.1	80	26
105	low	10	101.85	90	36
105	low	11	103.7	96	52
105	low	12	105.25	102	68
105	low	13	105.1	108	56
105	low	14	104.4	112	64
106	high	0	101.3	96	60
106	high	1	100.85	106	44
106	high	2	101.65	84	36
106	high	3	101.25	84	34
106	high	4	101.3	93	36
106	high	5	101.35	98	36
106	high	6	100.95	98	36
106	high	7	100.95	94	46
106	high	8	100.8	84	42
106	high	9	100.95	84	38
106	high	10	101.15	94	50
106	high	11	102	82	38
106	high	12	103.3	94	38
106	high	13	104.55	118	54
106	high	14	105.6	108	48
107	high	0	100.4	118	54
107	high	1	100.7	114	40
107	high	2	101.05	96	44
107	high	3	101.15	110	40

107	high	4	100.8	94	42
107	high	5	101.05	106	36
107	high	6	101.55	98	42
107	high	7	101.35	104	36
107	high	8	101.05	98	52
107	high	9	103.1	90	64
107	high	10	103.9	104	46
107	high	11	104.5	90	34
107	high	12	104.7	100	30
107	high	13	105.5	114	54
107	high	14	104.1	100	56
108	low	0	101	100	46
108	low	1	101.65	98	40
108	low	2	101.75	96	62
108	low	3	101.35	94	60
108	low	4	101.1	92	26
108	low	5	101.4	94	42
108	low	6	101.55	92	56
108	low	7	101.6	84	42
108	low	8	101	96	34
108	low	9	101.5	84	40
108	low	10	101.7	86	24
108	low	11	100.8	76	32
108	low	12	100	74	28
108	low	13	100.35	74	26
108	low	14	99.4	72	28
138	high	0	100.6	100	43
138	high	1	100.95	92	44
138	high	2	101.3	80	47
138	high	3	100.95	96	40
138	high	4	100.75	94	38
138	high	5	100.55	66	38
138	high	6	100.7	60	36
138	high	7	101.3	70	38
138	high	8	102.9	86	46
138	high	9	102.95	92	46
138	high	10	103.9	88	60
138	high	11	104.8	106	58
138	high	12	105	110	80
138	high	13	105.1	134	84
138	high	14			
