# Supplementary material for: Development of septic polysynovitis and uveitis in foals experimentally infected with Rhodococcus equi
Source: PLoS One. 2018 Feb 7;13(2):e0192655. doi: 10.1371/journal.pone.0192655 (PMC5802921; doi:10.1371/journal.pone.0192655)
Supplement: S2 Table — Foals were infected with a high inoculum (1 × 10^8^ CFU; n = 16) or a low inoculum (1 × 10^7^ CFU; n = 12) of virulent R. equi. (PDF) [file pone.0192655.s002.pdf]

Supplementary table 2. Occurrence of uveitis or polysynovitis, percentage of affected lung, culture results, and fluid protein concentrations. Foals were infected with a high inoculum (1 × 10^8 CFU; n=16) or a low inoculum (1 × 10^7 CFU; n=12) of virulent *R. equi*.

Foal ID	Challenge dose	Lung area (%)	Uveitis	Polysynovitis	Aqueous protein (mg/gL)	Synovial fluid Protein (g/dL)	Aqueous culture	Joint fluid culture	Bacteria seen histopathology[a]
77	high	33.5		1		3.1		1	
78	low	29.0	1	1	816	3.25	0	1	1
79	high	44.4	1	1	2320	2.65	0	1	1
80	high	46.5		1		2.65		1	
82	high	39.9	1	1	817.5	3.6	1	1	
84	high	28.1	1	1	1826.5	3.35	1	0	
87	high	26.7	1	1	2332	3.4	0	1	0
88	low	33.5	1	1	1278.5	2.5	0	0	1
89	high	26.3	0	1	35	3.35	1	0	1
90	high	0.0	0	0	55.5	1.5	0	0	0
91	low	0.0	0	0	13.5	1.5	0	0	0
92	low	12.5	0	0	37	2.05	0	0	0
93	high	40.5	1	1	2320.5	3.25	0	1	0
94	high	40.1	1	1	2245.5	3.9	1	1	
96	low	19.7	1	1	834	2.9	0	0	1
97	low	20.1	1	1	545	3.25	1	1	1
98	high	21.2	0	1	253	1.65	1	1	1
99	low	0.5	0	0	143.5	2.25	0	0	0
100	low	18.3	0	0	74.5	2.1	1	1	0
101	low	0.3	0	0	16	1.65	1	1	0
102	low	3.7	0	1	75	2.6	1	0	0
103	high	2.9	0	0	296	1.2	0	0	0
104	high	34.2	1	1	1999	2.75	0	1	0
105	low	22.5	1	1	746.5	3.75	0	0	1
106	high	27.1	1	1	2344	3.3	0	0	1
107	high	45.6	1	1	1667	2.65	1	1	1
108	low	13.0	0	0	136	1.65	1	0	0
138	high	51.1		1		2.3		0	

0: no or negative; 1: yes or positive; [a] histopathology of the synovial membrane
